# 

**Published:** 1896-01

**Authors:** 


### CONTENTS.

### *EDITORIAL.*

PAGE

Arsenicum-album, . . . . . . . . . . . . . . . . . . . . . . . . . . . . . . . . . . 1

### *MISCELLANEOUS.*

*The Organon* and Materia Medica Club of the Bay Cities of California, . . . . . . . 3

Proceedings of the Society of Electro-Therapeutists, . . . . . . . . . . . . . . . . . 12

The Truth Shall Make us Free—Third Article. J. H. Allen, M. D., . . . . . . . . . . 18

Antimonium-tartaricum. Clinton Enos, M. D., . . . . . . . . . . . . . . . . . . . . 22

Lachesis—Its Origin and Pathogenetic Effects. Prof. W. E. Leonard, M. D., . . . . 27

Lachesis to the Rescue. Jos. Fitz-Mathew, M. D., . . . . . . . . . . . . . . . . . . 30

Formica-rufa. J. W. Thomson, M. D., . . . . . . . . . . . . . . . . . . . . . . . . . 32

The Application of the Materia Medica. C. M. Boger, M. D., . . . . . . . . . . . . . 33

Imagination in Medicine. Stuart Close, M. D., . . . . . . . . . . . . . . . . . . . . 35

The Cause. A. A. Waterhouse, M. D., . . . . . . . . . . . . . . . . . . . . . . . . . 39

The Hahnemann Association of New York, . . . . . . . . . . . . . . . . . . . . . . 40

Diphtheria and Anti-Toxin, . . . . . . . . . . . . . . . . . . . . . . . . . . . . . . 41

Cases. Dr. Kunkel, of Kiel. Translated by A. McNeil, M. D., . . . . . . . . . . . . 43

The New Society of Hahnemannians, . . . . . . . . . . . . . . . . . . . . . . . . . 47

Vomiting of Fluids but not of Solids, . . . . . . . . . . . . . . . . . . . . . . . . . 48

Truth Stranger than Fiction, . . . . . . . . . . . . . . . . . . . . . . . . . . . . . . 49

Anti-Toxin Notes, . . . . . . . . . . . . . . . . . . . . . . . . . . . . . . . . . . . 49

### *BOOK NOTICES.*

The Non-Heredity of Inebriety, by Leslie E. Keeley, M. D., LL.D., . . . . . . . . . . 50

A Repertory of Hering's Guiding Symptoms, by C. B. Knerr, M. D., . . . . . . . . . . 51

The Standard Dictionary of Funk & Wagnalls, . . . . . . . . . . . . . . . . . . . . 53

The Archives of Pediatrics, . . . . . . . . . . . . . . . . . . . . . . . . . . . . . . 53

Diseases of Rectum and Anus, . . . . . . . . . . . . . . . . . . . . . . . . . . . . . 54

Color-Vision and Color-Blindness, . . . . . . . . . . . . . . . . . . . . . . . . . . . 54

Urinalysis, . . . . . . . . . . . . . . . . . . . . . . . . . . . . . . . . . . . . . . . 54

Delicate, Backward, Puny, and Stunted Children, . . . . . . . . . . . . . . . . . . . 55

Annales D'Oculistique, . . . . . . . . . . . . . . . . . . . . . . . . . . . . . . . . . 56

Appendicitis, . . . . . . . . . . . . . . . . . . . . . . . . . . . . . . . . . . . . . . 57

### *NOTES AND NOTICES.*

Electrolysis for the Surgical Treatment of Strictures — The Hahnemann Club of Philadelphia, Pa.—Dr. Bushrod W. James—Dr. J. H. Allen—The National Association of Physicians and Surgeons—Advertising Axioms, . . . . . . . . 57

Fun for Doctors, . . . . . . . . . . . . . . . . . . . . . . . . . . . . . . . . . . . . 60

